# Event-Related Potential Measures of a Violation of an Expected Increase and Decrease in Intensity

**DOI:** 10.1371/journal.pone.0076897

**Published:** 2013-10-15

**Authors:** Margaret Macdonald, Kenneth Campbell

**Affiliations:** School of Psychology, University of Ottawa, Ottawa, Ontario, Canada; University of Salamanca- Institute for Neuroscience of Castille and Leon and Medical School, Spain

## Abstract

Unexpected physical increases in the intensity of a frequently occurring “standard” auditory stimulus are experienced as obtrusive. This could either be because of a physical change, the increase in intensity of the “deviant” stimulus, or a psychological change, the violation of the expectancy for the occurrence of the lower intensity standard stimulus. Two experiments were run in which event-related potentials (ERPs) were recorded to determine whether “psychological” increments (violation of an expectancy for a lower intensity) would be processed differently than psychological decrements (violation of an expectancy for a higher intensity). Event-related potentials (ERPs) were recorded while subjects were presented with auditory tones that alternated between low and high intensity. The subjects ignored the auditory stimuli while watching a video. Deviants were created by repeating the same stimulus. In the first experiment, pairs of stimuli alternating in intensity, were presented in separate increment (H-L…H-L…H-**H**…H-L, in which H = 80 dB SPL and L = 60 dB SPL) and decrement conditions (L-H…L-H…L-**L**… L-H, in which H = 90 dB SPL and L = 80 dB SPL). The paradigm employed in the second experiment consisted of an alternating intensity pattern (H-L-H-L-H-**H**-H-L) or (H-L-H-L-**L**-L-H-L). Importantly, the stimulus prior to the deviant (the standard) and the actual deviants in both increment and decrement conditions in both experiments were physically identical (80 dB SPL tones). The repetition of the lower intensity tone therefore acted as a psychological rather than a physical decrement (a higher intensity tone was expected) while the repetition of the higher intensity tone acted as a psychological increment (a lower intensity tone was expected). The psychological increments in both experiments elicited a larger amplitude mismatch negativity (MMN) than the decrements. Thus, regardless of whether an acoustic change signals a physical increase in intensity or violates an expected decrease in intensity, a large MMN will be elicited.

## Introduction

Detection of a change in any feature of an auditory stimulus plays a critical role in the survival of mammalian species, providing information regarding the presence and position of potential predators or mating partners. Subtle changes in intensity are however especially important for the signalling and recognition of emotional expression. In this regard, an increase in the intensity of auditory input may signal the approach of an organism while, on the other hand, a decrease in intensity may signal the retreat of an organism. In support of this notion, studies have shown that an increase in stimulus intensity is experienced as “looming” or approaching and may elicit a fear response in animals and a decrease in intensity is experienced as “fading” or retreating [1]–[3].

The brain has evolved a set of mechanisms to detect such change and redirect attention away from ongoing cognitive activity and toward this potentially more relevant auditory input. Such operations do have the critical advantage that they allow the organism to detect highly relevant auditory stimuli and to subsequently take appropriate action even though attention may be directed elsewhere. On the other hand, the interruption of ongoing cognitive activities does come at a cost, distraction. Frequent and inappropriate distraction is a major symptom of many neurological and psychiatric disorders.

The classic Näätänen [4] model describes the types of processing required to detect a change in the features of an acoustic signal. The model has undergone revision recently [5], while other models including a regulatory extraction model [6] and a predictive coding model [7] have also been developed. There is a good deal of overlap among the models and most assume an encoding and memory storage of the regularities in previous “standard” stimulation and a comparison of the features of an incoming stimulus with those stored in memory. In the Näätänen model, if these features match, further processing is deemed redundant and thus ceases. If any feature (e.g., frequency, intensity, duration) fails to match, the change detection system is activated. Unique to this model is the use of event-related potentials (ERPs) to provide a means to monitor the extent of processing when the subject is not actively attending to the auditory channel. The detection of a stimulus regularity that signals change from the past is reflected by a fronto-centrally maximum negative-going ERP component, the mismatch negativity (MMN) occurring approximately 150–250 ms following the onset of the deviant auditory stimulus and is often larger over right than left frontal regions. The output of this system varies in proportion to the extent of change. If the magnitude of change reaches a certain variable threshold, an interrupt is sent to the central executive controlling the allocation of cognitive resources, resulting in a halting of ongoing activities and a switching of attention to the auditory channel. This process of redirection has been called “attention capture” and is indexed by a different and later ERP component, the P3a, a centro-frontally maximum positivity occurring 200–300 ms following the onset of a deviant auditory stimulus [8].

The MMN is often recorded using the so-called “oddball” paradigm. This paradigm consists of a series of physically identical frequently occurring repetitive “standard” stimuli that are periodically changed to a “deviant” stimulus whose physical features differ in some way from the preceding standard series. Muller-Gass et al [9] employed an oddball paradigm to examine the influence of a change in auditory intensity on attention capture. Participants were presented with a frequently occurring 80 dB SPL auditory “standard” on 85% of trials and either a 90 dB SPL increment or 60 dB SPL decrement “deviant” on the remaining trials. The 90 dB SPL and 60 dB SPL deviants represent approximately equivalent changes in acoustic energy relative to the 80 dB SPL standard. Subjects’ attention was diverted to a continuous visual multiple moving object task and thus the auditory stimuli were irrelevant and to-be-ignored. The MMN is best observed in a difference wave computed by subtracting the ERPs elicited by the standard from those elicited by the deviant. The subtraction procedure isolates processing that is unique to the deviant by removing processing that is common to both the standard and deviant. An MMN and a small amplitude P3a were evident in the decrement difference wave in this study, while a much larger negativity, followed by a large P3a were observed in the increment difference wave. The larger amplitude of the P3a elicited in response to the increment in this study lends support to the notion that large increases in stimulus intensity are more disruptive than decreases [10], [11].

The large negativity that was evident in the difference wave following presentation of the increment was probably not an unambiguous MMN, however. This is because in the Näätänen model [4] increments in intensity also result in increased activation of a second, different system, the transient detection system, in addition to the change detection system. The output of the transient detection system, as indexed by the N1 ERP, varies in proportion with the energy (or intensity) of the stimulus. A deviant, whose intensity is higher than that of the standard, will therefore elicit a larger N1 than the standard and also an MMN; the N1 and MMN thus summate both temporally and spatially. Because the negativity that is observed on the scalp recording is a composite N1+MMN, it is called a deviant-related negativity (DRN). By contrast, a deviant whose intensity is lower than the standard will elicit a smaller N1 but also again, an MMN, because it also signals change. Thus, the additional negativity elicited by a decrement deviant can only be attributed to increased activation of the change detection system.

The obtrusiveness of an intensity increment can even be observed in an unconscious state, natural sleep. Macdonald et al [11] employed an oddball paradigm to examine the differences in the processing of intensity increments and decrements during the waking and sleeping states. Subjects were presented with a similar oddball sequence consisting of frequently occurring 80 dB SPL standard tones and rare 90 dB SPL increment or 60 dB SPL decrement deviants. The results observed during the waking state mirrored those of the Muller-Gass et al. study [9], with the increment resulting in a larger negativity than the decrement, and both deviants successfully eliciting a P3a. The increment deviant was successful in eliciting a negativity corresponding to the latency and scalp distribution of a DRN and a P3a during the REM stage of sleep. The decrement deviant failed to elicit either of these ERPs. During non-REM sleep, neither the increment nor decrement elicited a DRN. The findings from both studies appear to indicate that increments in intensity are much more salient than decrements of equal magnitude, and are more likely to passively capture the attention of an observer.

Recently, researchers have begun to use more complex patterns rather than simple oddball paradigms to study the change detection process [12]. The change detection system has been shown to be sensitive to violations in both concrete and abstract rule-based acoustic patterns [13]–[18]. Alain et al [19] and Sculthorpe et al. [20] employed a concrete rule-based pattern consisting of high and low frequency alternating tones (A-B-A-B-A-B-A-B), with deviants created by the repetition of a tone within the sequence (A-B-A-B-A-B-**B-**B-A). The deviant in this paradigm was thus physically identical to the stimulus preceding it (i.e., the standard). A highly consistent finding in the neurophysiological literature is that the repetition of the same, identical rapidly-presented stimulus will result in a reduction of the response because of refractory and possibly habituation processes. This was not the case in the Alain et al. and Sculthorpe et al. studies. The deviants in both studies elicited a larger negativity, the MMN, than the physically identical standard stimulus that preceded it. This is because the deviant violates the *expectancy* for a high or low frequency tone; it thus signals a psychological rather than a physical change.

Macdonald and Campbell [21] employed an alternating pattern with the intensity of the auditory tones alternating rather than their frequency. A sequence of 1000 Hz tones followed a standard, rule-based alternating high-low intensity pattern (H-L-H-L-H-L). This standard pattern was periodically interrupted by a deviant created by repeating one of the stimuli in the sequence (e.g., H-L-H-L-**L-**L-H). The repetition of the high intensity stimulus represented what Macdonald and Campbell [21] called a relative, *psychological* increment compared to what the alternating rule would have predicted (the low intensity). Because the deviant was created by the repetition of a physically identical stimulus (the high intensity) that did not differ in intensity relative to the preceding standard, it should not have resulted in increased activation of the transient detector system. Similarly, the repetition of the low intensity stimulus acted as a relative, *psychological* decrement compared to what the alternating rule would have predicted (the high intensity). Thus, the alternating pattern paradigm employed in this study allowed for direct comparison of the processing of increment and decrement stimuli within the change detection system, circumventing additional activation of transient detector activation and the overlapping N1 component. In different conditions, the intensity difference between the low and high intensity tones was 3 dB (75 dB SPL and 78 dB SPL), 9 dB (72 dB SPL and 81 dB SPL) or 27 dB (63 dB SPL and 90 dB SPL). The repetition of the lower intensity tone (L-L), the psychological “decrement” deviant, thus represented a 3, 9, or 27 dB violation of the expected intensity increment (L-H) rule, while the high intensity repetition (H-H), the psychological “increment” deviant, represented a 3, 9, or 27 dB violation of the expected intensity decrement (H-L) rule. A large MMN was elicited only when the separation between the low and high intensities was 27 dB. Importantly, this MMN peaked significantly earlier and its amplitude was significantly larger following presentation of the psychological increment. The results of this study would seem to indicate that psychological increments are still more salient than decrements even though the repetition of the high intensity stimulus (the deviant) would result in less activation of the refractory-based transient detector system.

There remains a problem with this conclusion. Jacobsen et al. [22] suggest that in the ideal study, all standards and deviants should be physically identical. In the Macdonald and Campbell study [21], while the standard and psychological increment deviant (both 90 dB SPL) and the standard and psychological decrement deviant (both 63 dB SPL) were physically identical, the two deviants were nevertheless physically different (90 vs 63 dB SPL). The repetition of a 63 dB SPL stimulus (the psychological decrement) may not be experienced in the same way as the repetition of a 90 dB SPL stimulus (the psychological increment). In order to address this issue, the present study consists of two experiments again employing an alternating high and low intensity pattern, but now, the standards and also the increment and decrement deviants will be physically identical.

## Experiment 1: Alternating Paired Pattern

The MMN can also be elicited in a somewhat different “paired” pattern to examine the influence of psychological intensity change. Paavilainen et al. [17] presented two brief duration tones separated by 50 ms followed by a 500 ms pause. In an ascending condition, the standard pattern followed the rule that the intensity of the second tone in the pair was 7 dB higher than the first. Deviation in the sequence occurred when the second tone descended in intensity. The presentation of the lower intensity deviant violated the rule that the second stimulus is higher in intensity than the first, thus also representing a psychological decrement in intensity. However, the deviant was physically lower in intensity. In a second descending condition, the presentation of descending pairs was the standard pattern that was sporadically interrupted by an ascending deviant pair. Again, this represented both a psychological and a physical increment in intensity. Both the increment and decrement deviants were successful in eliciting DRNs. Unfortunately, the authors did not examine differences between the ERPs elicited in response to the increment and decrement deviants. In the decrement condition the DRN was probably only an MMN, solely reflecting increased activation of the change detection system. The DRN elicited by the increment deviant, however, may have reflected a composite DRN, as a result of increased activation in both the transient and change detection systems.

Experiment 1 of the present study employed a paired alternating pattern paradigm to compare the processing of psychological increment and decrement stimuli within the change detection system, while controlling for physical differences in the two types of deviants. The study was designed so that two alternating paired pattern conditions were presented, so that an identical 80 dB SPL tone served as a standard and also as both decrement and increment deviants. Any differences in processing between the psychological increment and decrement could thus be attributed to physical differences between the repeated deviants.

### Methods

#### Subjects

Ten young adults (4 males) between the ages of 20 and 29 years (Mean = 25.4 years) volunteered to participate in this experiment. None reported a history of neurological disorder or auditory impairment. Written informed consent was obtained prior to the experiment and subjects received an honorarium for participation. The study was conducted according to the guidelines of the Canadian Tri-Council (Health, Natural and Social Sciences) on ethical conduct involving human subjects. Procedures were approved by the University of Ottawa Research Ethics Board.

#### Procedure & Stimuli

An InstEP system was used for the presentation of stimuli, and the collection and analyses of the ERP data. Subjects were seated in a sound-attenuated room during the EEG recording sessions. They were instructed to attend to a silent, subtitled film while auditory stimuli were presented via headphones. Two different types of auditory patterns were presented to subjects. The first, the increment condition, consisted of an alternating high-low intensity “standard” pattern with stimuli presented in pairs (H-L…H-L…H-L). The second consisted of an alternating paired low-high intensity (L-H…L-H…L-H) pattern. Deviants were created by the repetition of the of the second stimulus in the standard pair (e.g., H-L…H-L…H-**H**…H-L in the case of the increment or L-H…L-H…L-**L**…L-H in the case of the decrement). The deviants thus represented a psychological change rather than a physical change in intensity relative to the immediately preceding stimulus. In the psychological increment condition, a standard 80–60 dB SPL pair was presented. On 12.5% of trials, this was changed to an 80–**80** dB SPL deviant pair. In this case, because the pattern rule establishes that the second member of the pair should be low intensity (i.e., 60 dB SPL), the presentation of the 80 dB SPL deviant thus acts as a psychological increment (its intensity was higher than what would be expected by the rule). In the psychological decrement condition, a standard 80–90 dB SPL pair was presented. The violation of the pattern was again created by presenting the same 80–**80**dB SPL pair, on 12.5% of trials. Because the pattern rule establishes that the second member of the pair should be high intensity (i.e., 90 dB SPL), the presentation of the 80 dB SPL deviant acts as a psychological decrement. Importantly, the deviants in the psychological increment and psychological decrement conditions were physically identical (i.e., 80–80 dB SPL pairs). Still, in this paradigm the standard stimulus pair (e.g., 80–60 or 80–90 dB SPL) was physically different from the deviant pair (80–80 dB SPL). Two oddball conditions were therefore run in which the standard was always an 80–80 dB SPL pair. The deviant pair was either 80–90 dB SPL in the oddball increment condition (L-L…L-L…L-l…L-H…L-L) or 80–60 dB SPL in the oddball decrement condition (H-H…H-H…H-H…H-L…H-H). The deviant occurred with the same probability of occurrence as in the paired alternating paradigm. Presentation of the oddball conditions served to provide a standard (80–80 dB SPL) that resolved the problem of having physically different standard pairs in the increment and decrement pattern conditions. The standard pairs in the oddball increment and decrement conditions were physically identical (80–80 dB SPL) and they were also physically identical to the psychological increment and decrement deviants in the paired pattern paradigm. The ERPs to the standard 80–80 dB SPL pairs in the oddball conditions were then subtracted from the physically identical 80–80 deviant pairs in the psychological increment and psychological decrement conditions.

Separate increment and decrement conditions were presented corresponding to each of the two sequences (alternating pattern and oddball). Thus, each participant was presented with auditory patterns corresponding to four separate conditions: psychological decrement (L-H…L-H…L-**L**…L-H), psychological increment (H-L…H-L…H-**H**…H-L), oddball decrement (H-H…H-H…H-**L**…H-H) and oddball increment (L-L…L-L…L-**H**…L-L). Stimuli in all conditions were presented in pairs with a within-pair SOA (offset-to-onset) of 100 ms and a between-pair SOA of 1800 ms. All stimuli had a frequency of 1000 Hz and a total duration of 55 ms (5 ms rise/fall). Within each condition, the standard and deviant stimuli were presented in pseudo-randomized order, with the constraint that a deviant was followed by a minimum of 3 standard pairs. Similarly, a minimum of 3 standard pairs were presented at the beginning of each condition prior to the occurrence of a deviant to allow for the standard pattern to be established in memory. Three blocks of each of condition were presented (i.e., a total of twelve blocks), with each block containing a total of 160 stimulus pairs. Subjects were permitted a brief break after each block. The conditions within each block were presented in random order. Total testing time was therefore about 90 minutes.

#### Performance task

The amplitude of the MMN is affected by perceptibility of the deviant. Deviants that are easy to perceive elicit a larger amplitude and shorter latency MMN. The perceptibility of the increment and decrement deviants was therefore determined in a separate behavioural discrimination task following the physiological recording sessions. The same stimulus pairs were used. A single block of stimuli was presented for the psychological increment and the decrement conditions. Subjects were instructed to press a keyboard button as quickly and accurately as possible upon detection of a violation of the standard L-H or H-L pattern (i.e., a repetition of the same stimulus). Both accuracy of detection (“hits”) and reaction time (RT) were recorded. A response had to be made within 1000 ms after stimulus onset in order to be considered a hit.

#### Physiological recording

The EEG was recorded from 10 scalp sites representing frontal (F3, Fz, F4), central (Cz, C3, C4), parietal (Pz), temporal (T7, T8), and occipital (Oz) areas of the scalp using silver/silver chloride electrodes attached to an electrode cap (Electro-Cap International Inc., Eaton, OH). Two additional channels were recorded from individual electrodes placed on the left and right mastoids (M1, M2). The nose served as a reference. A true N1 and MMN will invert in polarity (i.e., be recorded as a positive potential) at the mastoids when a nose reference is used. Vertical eye movements and blinks were recorded from electrodes placed at the infra- and supra-orbital ridges of the left eye. A horizontal EOG was recorded from electrodes placed at the outer canthus of each eye. The ground electrode was located between the Fz and FPz sites. Inter-electrode impedances were kept below 5 kΩ.

The physiological signals were digitized continuously at a 256 Hz sampling rate and stored on hard disk. The high frequency filter was set at 35 Hz and the time constant at 2 s. Offline, an inverse FFT high digital filter set at 15 Hz was applied to the data. Eye movements and blinks were corrected using an algorithm operating in the time and frequency domains [23]. The participants’ continuous data were partitioned into 600 ms epochs beginning 100 ms before onset of the second stimulus in the pair and continuing for another 500 ms following it and baseline corrected. Epochs containing EEG that exceeded ±100 µV were considered to be artefact and excluded from further analyses. Individual participant’s data were then averaged according to electrode site and stimulus type (oddball standards (i.e., 80–**80 **dB SPL) and pattern increment and decrement deviant (i.e., 80–**80 **dB SPL)). The second stimulus within a pair of repetitive stimuli was considered to be the deviant within a condition. The three pairs of stimuli presented immediately following the deviants were omitted from averaging to allow reformation of the memory for the standard sequence pattern required for change from this pattern to be detected. Similarly, the first three pairs of stimuli in each condition were also omitted from averaging (a memory for the standard pattern would not yet have been formed).

#### Quantification of the MMN

The standard stimuli in the oddball conditions (80–80 dB SPL) were subtracted from the physically identical deviant occurrences of these pairs in the psychological increment and decrement conditions in order to isolate processing that is unique to the detection of the deviants. Difference waves were computed by subtracting point-by-point the ERPs elicited by the auditory standard from the deviant pairs at each electrode site.

The MMN and P3a were initially identified in the grand average (average of all subjects’ averages) difference wave. In individual subjects, the MMN and P3a were quantified as the mean of all data points within ±20 ms of the peak in the grand average.

There are problems with the use of common statistical procedures. Macdonald and Campbell [21] indicated that the MMN was much reduced and the P3a absent following presentation of a psychological decrement deviant. The observation of a significant difference in the amplitude of a component between conditions cannot be used as evidence that it was in fact elicited in both conditions. To avoid these ambiguities, it is necessary to first demonstrate that significant MMN and P3a components were indeed elicited in the different conditions. Confidence intervals were computed for the amplitude of the MMN and P3a. This procedure determined the probability that the mean amplitude value fell within an upper or lower range of the pre-stimulus baseline zero voltage level. Thus, in the case of the MMN, when the lower limit of a confidence interval was significantly less than 0 µV, (i.e., was negative-going), the interval was considered to contain a significant negativity. This procedure is equivalent to computing a *t*-test between the standard and deviant waveforms [24]. Because a negative directionality was predicted in the case of the MMN and a positive directionality was predicted in the case of the P3a, one-tailed tests of significance (p<0.05) were applied to the confidence intervals. To restrict the likelihood of chance findings, the negativity had to conform to the usual latency (100–250 ms) and scalp distribution (fronto-central maximum, inversion in polarity at the mastoids) of the MMN, while the positivity had to conform to the usual latency (200–350 ms) and scalp distribution (centro-frontal maximum) of the P3a.

These data were subjected to one-tailed t-tests comparing the amplitude of responses elicited by the increment to those elicited by the decrement. One tailed t-tests were applied because previous findings have indicated that the increment would elicit a larger amplitude and shorter latency MMN than the decrement [21]. Performance data (i.e., hit rates, reaction times) were also analyzed via one-tailed t-tests. In the case of the performance data, the use of one-tailed tests of significance would increase the likelihood of findings differences in perceptibility of the increment and decrement deviants.

### Results

#### Performance data

Subjects’ ability to identify the psychological increment and psychological decrement deviants in the alternating pattern paradigms did not significantly differ, t<1. Reaction times were, however, found to be significantly faster when the 80–80 deviant pair represented a psychological increment (i.e., from the 80–60 dB SPL standard), as compared to a psychological decrement (i.e., from the 80–90 dB SPL standard), t(9) = 6.14, p<0.01.

#### Physiological data

**Figure 1:**
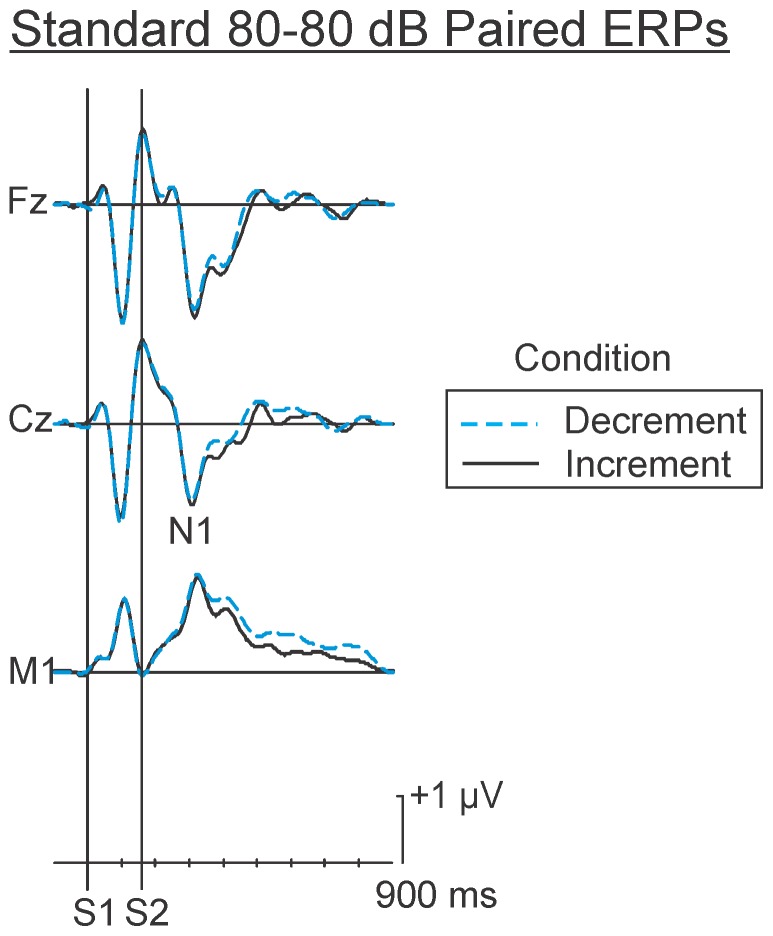
Grand average “raw” ERPs elicited by the standard pairs in the oddball conditions. In this and all other figures, positivity at the scalp relative to the reference is indicated by an upward deflection. The first 80–80 dB SPL standards in the increment and decrement conditions are superimposed. The morphology of these ERPs are essentially equivalent. Both include an N1s occurring approximately 100 ms followed by a positive deflection and another N1 approximately 140 ms following the onset of the second. These standard ERPs were subsequently subtracted from the ERPs elicited by the deviant 80–80 dB SPL pairs in the psychological increment and decrement conditions in order to isolate processing associated with the change detection process.

This study examined differences in the MMN that were elicited by the psychological decrement and increment deviants in different conditions. Because the MMN is measured in a difference wave (i.e., deviant minus standard ERPs), an assumption is made that the processing of the standards is constant across the two conditions. Any differences observed in the difference wave could therefore only be attributed to the processing of the psychological detection of change (i.e., the processing of the deviant). The assumption that processing of the standards was constant in the two conditions was tested. Figure 1 depicts the ERPs elicited by the same standard auditory stimulus pairs (80–80 dB SPL) in the increment and decrement oddball conditions. Two distinct negative deflections are apparent at about 100 and 310 ms. The first negative peak is the N1 to the initial stimulus (80 dB SPL), while the second negative deflection is the N1 to the second 80 dB SPL stimulus in the standard pair. As may be observed, the standard ERPs are very similar in both the increment and decrement oddball conditions, F<1 in all intervals.

**Figure 2:**
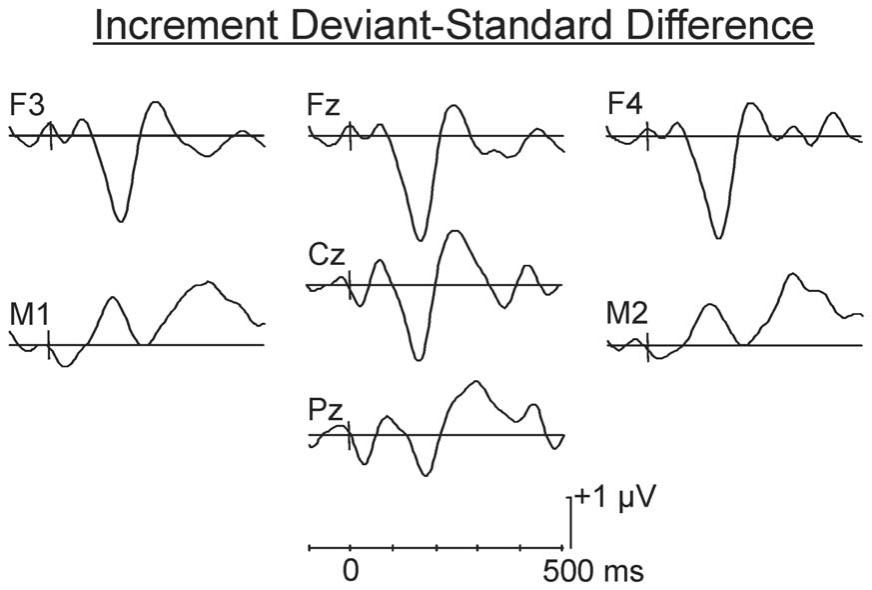
Grand average difference waves in the psychological increment condition. Difference waves were created by subtracting frequently occurring 80–80 dB SPL standard pairs in the oddball decrement condition (80–80…80–80…80–60…80–80) from deviant occurrences of these pairs in the psychological increment condition (80–60…80–60…80–**80**…80–60). The onset of the second stimulus (when deviance from the standard pattern occurs) is set at 0 ms in this figure. A large amplitude MMN was evident over fronto-central areas of the scalp between 150 and 200 ms following presentation of the psychological increment. The MMN inverted in polarity at the mastoids. However, this inversion failed to reach significance at either the M1 or M2 electrode sites. A centrally maximum non-significant P3a-like wave is evident at approximately 280 ms.

The difference waves corresponding to the psychological increment condition are presented in Figure 2. The onset of the second stimulus in the pair (the deviant) occurs at 0 ms in this figure. As may be observed, the deviant in the psychological increment condition (80–60…80–60… 80–**80**…80–60) elicited a large (M = −3.16 SD = 2.40 µV) fronto-central MMN, peaking at approximately 195 ms. Confidence interval testing revealed that the amplitude of this component differed significantly from the zero-voltage baseline (p<0.05). The MMN inverted in polarity at the mastoids. A centrally maximum P3a-like wave, peaking at approximately 280 ms, was evident in the difference wave. However, this positivity failed to reach significance as determined by confidence interval testing.

**Figure 3:**
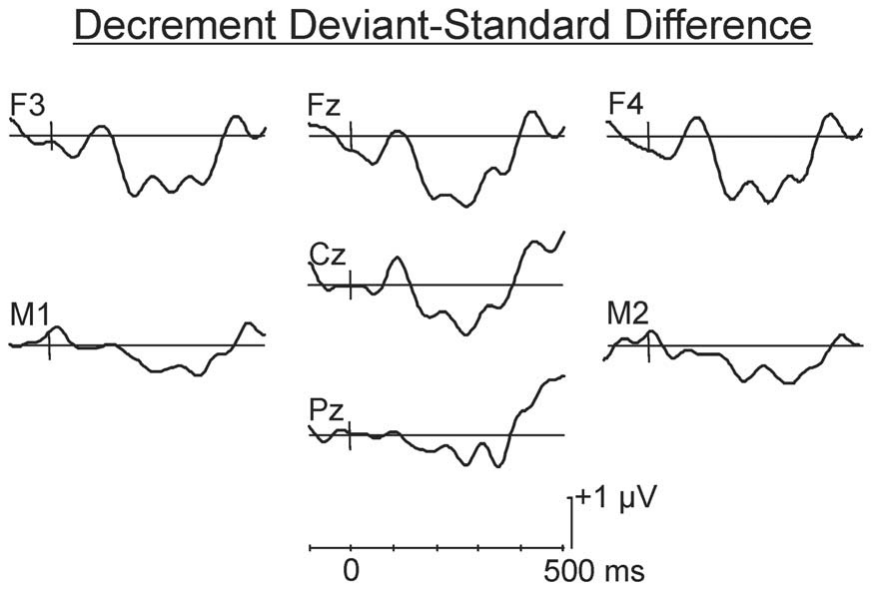
Grand average difference waves in the psychological decrement condition. Difference waves were created by subtracting frequently occurring 80–80 dB SPL standard pairs in the oddball increment condition (80–80…80–80…80–90…80–80) from deviant occurrences of these pairs in the psychological decrement condition (80–90…80–90…80–**80**…80–90). Again, the onset of the second (deviant) member of the pair is set at 0 ms in this figure. The psychological decrement elicited a smaller and relatively delayed (peaking between 200–250 ms) but significant MMN. The small inversion of the mastoids was not significant. No P3a-like positivity was present.

The difference waves corresponding to the deviant in the psychological decrement condition (80–90…80–90…80–**80**…80–90) are illustrated in Figure 3. In this condition, the psychological decrement elicited a smaller MMN (M = −1.47 µV, SD = 1.37 µV ), peaking at approximately 220 ms. Confidence interval testing indicated that this negativity differed significantly from zero-voltage baseline (p<0.05). An inversion of the MMN was not apparent at the mastoids. Similarly, a P3a was not apparent.

A one-tailed t-test was run on the Fz data (where the MMN was largest) to compare the MMN in the psychological increment (80–60…80–60…80–**80**…80–60) and decrement (80–90…80–90…80–**80**…80–90) conditions. The amplitude of the MMN was significantly larger following presentation of the psychological increments, t(9) = 1.93, p<0.05. The latency of the MMN was also measured at its maximum peak in individual subjects. It was significantly earlier following presentation of the psychological increments, t(9) = 2.90, p<0.01. The MMN responses were larger in amplitude at F4 than F3 in both conditions; neither of these differences was however statistically significant.

### Discussion

Deviants in both the psychological increment and psychological decrement conditions were successful in eliciting MMNs that differed significantly from the zero-voltage baseline, as determined by confidence interval testing. Deviants in the psychological increment condition elicited larger amplitude and shorter latency MMNs than those in the psychological decrement condition. Thus, a more prominent MMN was elicited when the regular 80–60 dB SPL pattern was interrupted by an 80–80 dB SPL deviant pair (i.e., a psychological increment). On the other hand, a smaller (but significant) MMN was elicited when the regular 80–90 dB SPL pattern was interrupted by the presentation of the same 80–80 dB SPL deviant pair (i.e., a psychological decrement). The deviant in the psychological increment condition also elicited a positivity, corresponding to the typical scalp-distribution and latency parameters of a P3a, but this positivity was not significantly different from the zero voltage baseline. The deviant in the psychological decrement condition did not elicit any P3a-like potential. Importantly, in contrast to the deviants employed in the Macdonald and Campbell study [21], the psychological increment and decrement were physically identical. The larger MMN observed in the psychological increment relative to the decrement condition cannot therefore easily be attributed to physical differences between the stimuli. When subjects were asked to detect the deviants, accuracy of detection (hit rate) did not differ for the psychological increments and decrements. RTs were however faster following presentation of the psychological increments. There is thus some evidence that the psychological increment was more easily perceived than the decrement.

Differences in perceptibility of the deviants might be related to the temporal integration of the standard pairs. Temporal integration occurs when the features of the present stimulus are added to (or “integrated” with) those of the preceding stimulus existing in sensory memory and is most likely to occur when stimuli are presented very rapidly (e.g., less than 200 ms) [25]. The time between the presentation of the two paired stimuli was 100 ms. The perception of the standard could thus be an integration of the 80 and 60 dB SPL pairs in the increment condition, and the 80 and 90 dB SPL pairs in the decrement condition. Because the same 80–80 dB SPL pair served as the deviant in both conditions, the integration of the pairs would not have varied across conditions. Thus in the psychological increment condition, in which the MMN was large, the occurrence of the 80–80 dB SPL deviant pair might be perceived as a *physical* increment in intensity relative to the integrated (80–60 dB SPL) standard pair. In the psychological decrement condition, in which the MMN was smaller, the occurrence of the same 80–80 dB SPL deviant pair might be perceived as a *physical* decrement in intensity relative to the integrated (80–90 dB SPL) standard pair. It is thus possible that MMN differences between the psychological increment and decrement could still be explained by physical differences in the perceived intensity and perhaps a resulting activation of the transient detection system.

## Experiment 2. Continuous Alternating Pattern

In order to remove the problem of possible temporal integration, a second experiment was run in which the occurrence of low and high intensity tones again alternated. They were not however presented in pairs. The stimuli in the psychological increment condition again consisted of 80 and 60 dB SPL tones, while those in the psychological decrement condition again consisted of 80 and 90 dB SPL tones. The time between the alternating stimuli was however 500 ms, well exceeding the minimal 200 ms time window usually associated with temporal integration [25]. The longer SOA should thus overcome the problem of feature integration and the possible increased activation of the transient detector system following presentation of the psychological increment. MMN differences between the increment and decrement deviants could thus be attributed to differential activation of only the change detection system.

### Methods

#### Subjects

Eleven young adults (6 males) between the ages of 19 and 33 years (Mean = 26.4 years) volunteered to participate in this study. None reported a history of neurological disorder or auditory impairment. Written informed consent was obtained prior to the experiment and subjects received an honorarium as compensation for participation. The study was again conducted according to the guidelines of the Canadian Tri-Council (Health, Natural and Social Sciences) on ethical conduct involving human subjects.

#### Procedure & Stimuli

Again, subjects were instructed to attend to a silent, subtitled film while auditory stimuli were presented via headphones. The auditory paradigm consisted of an alternating low-high intensity pattern (L-H-L-H-L-H) but in this experiment, the offset-to-onset SOA between the auditory stimuli was long, with a stimulus being presented every 500 ms. The alternating pattern was again violated by repeating the 80 dB SPL stimulus. Two different pattern violations were presented (L-H-L-H-L-**L-**L-H or L-H-L-H-**H-**H-L-H) in separate psychological increment and psychological decrement conditions. In the psychological increment condition, 80 dB SPL and 60 dB SPL tones were presented in an alternating pattern with the deviant created by the repetition of the higher intensity (80 dB SPL) tone (60-80-60-80-**80**-80-60). In the psychological decrement condition, 80 dB SPL and 90 dB SPL tones were presented in an alternating pattern with the deviant created by the repetition of the 80 dB SPL tone (90-80-90-80-**80**-80-90). The decrement and increment deviants each occurred 75 times within separate blocks of 600 stimulus pairings, making the total probability of a deviant repetitive pair.125 in each condition. The frequency of the tones was again 100 Hz and the total duration was 110 ms, (10 ms rise/fall time). The deviant pattern (H-H or L-L) was presented pseudo-randomly with the constraint that it followed a minimum of three and a maximum of 20 standard (L-H) stimulus pairings. Similarly, a minimum of 3 H-L or L-H standard alternations were presented at the beginning of each condition prior to the occurrence of a deviant repetitive stimulus to allow for the initial establishment of the standard pattern within memory. Two blocks of the psychological increment and psychological decrement conditions were presented to each participant (i.e., a total of four blocks) in random order. Subjects were permitted a brief break after each block. Total testing time was about 60 minutes. The auditory sequences were presented using E-Prime software (Psychology Software Tools Inc., Pittsburgh, PA) on a PC using Windows XP as an operating system.

#### Physiological recording

The number of scalp sites was increased to allow for the scalp distribution mapping of the MMN and possible P3a elicited by the psychological increments and decrements. EEG activity was recorded from 63 sites over frontal, central, parietal, temporal, and occipital sites using an active silver-silver chloride electrode system attached to an electrode cap (Brain Products, GmbH, Munich, Germany). Activity from the left and right mastoids was also recorded. An electrode was placed on the infra-orbital ridge of the left eye to record vertical eye movements. The tip of the nose was used as a reference for all channels. Inter-electrode impedances varied from 20 to 50 kΩ. A high filter was set at 500 Hz. The time constant was 2 s. The EEG was continuously digitized at a 250 Hz sampling rate and stored on hard disk for later analyses.

Offline, the data were reconstructed using Brain Products’ Analyzer2 software. The continuous data were digitally filtered using a high filter set at 15 Hz. EEG channels displaying high levels of noise were replaced by interpolating the data of the surrounding electrode sites [26]. Following offline inspection, the data of two participants were rejected. The first of these subjects’ data were discarded due to excessive levels of artefact across multiple channels, while the other subject failed to elicit either a sensory-related ERP (N1-P2) to the standard stimulus or an MMN in response to either deviant.

A vertical EOG was computed by subtracting activity recorded at FP1 from that at the lower EOG. A horizontal EOG was computed by subtracting the FT9 from the FT10 activity. Because of the large number of electrode placements, it was possible to use Independent Component Analysis (ICA) to identify eye movements and blinks that were statistically independent of the EEG activity [27]. These vertical and horizontal eye movements were then partialled out from the EEG.

The continuous EEG was segmented into discrete, 700 ms epochs including a 100 ms pre-stimulus baseline. The single trials epochs were subsequently baseline corrected. Any trials containing EEG activity exceeding ±100 µV on any channel were rejected from further analysis. The single epochs were sorted and averaged on the basis of electrode site and stimulus type (standard, decrement or increment).

#### Quantification of the MMN

As in the previous experiment, the MMN response was isolated in a difference wave representing the difference in processing between the standard and the deviant. The standards from the increment and decrement patterned paradigms (an 80 dB SPL tone that followed a 60 dB SPL or 90 dB SPL tone, respectively) were subtracted from the deviant occurrences of the 80 dB SPL stimuli (the repetition of an 80 dB SPL tone, 80–**80**). Thus, as in Experiment 1, in all conditions the standards and deviants were physically identical (i.e., an 80 dB SPL tone). The peak of the MMN and P3a components were again identified in the grand average. In individual subjects, they were quantified as the mean of all data points in the ±20 ms interval surrounding the peak.

### Results

**Figure 4:**
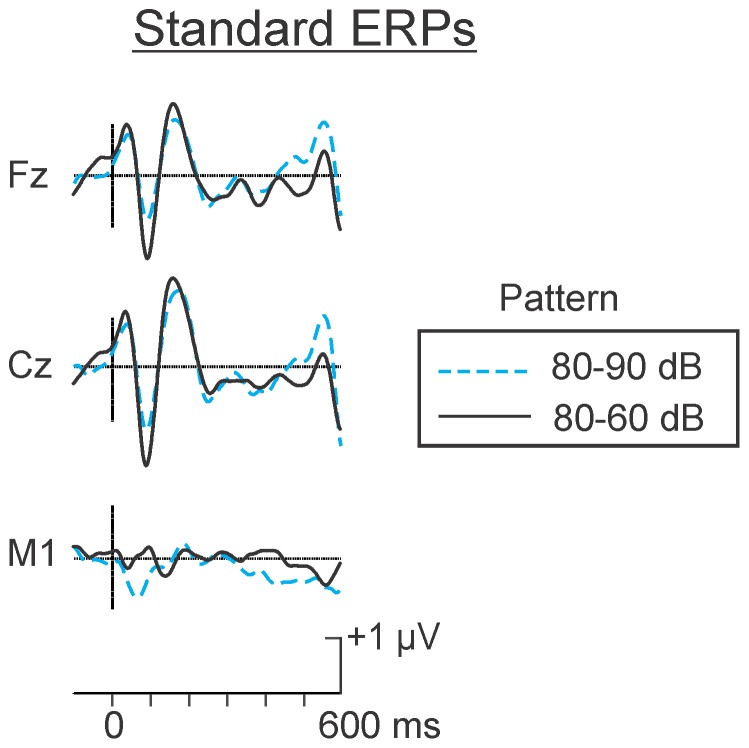
Grand average “raw” ERPs elicited by the same standard 80 dB SPL tones in the psychological increment and decrement conditions. ERPs corresponding to the standards in the two conditions are superimposed in the figure. N1, peaking at about 100(60 dB SPL) tone in the decrement condition but alternated with a higher intensity (90 dB SPL) tone in the increment condition.

In the present experiment, it was expected that the MMN observed in the difference wave would be larger in the increment condition. Again, however it is possible that the larger negativity in the difference wave would not be a result of an MMN to the deviant but rather of a smaller N1 to the standard. The raw waveforms corresponding to the grand averages of the 80 dB SPL standards in the psychological increment and psychological decrement conditions are represented in Figure 4. The N1 to the same 80 dB SPL standard stimulus was in fact larger in the increment (80–60 dB SPL pattern) condition than in the decrement (80–90 dB SPL pattern) condition.

**Figure 5:**
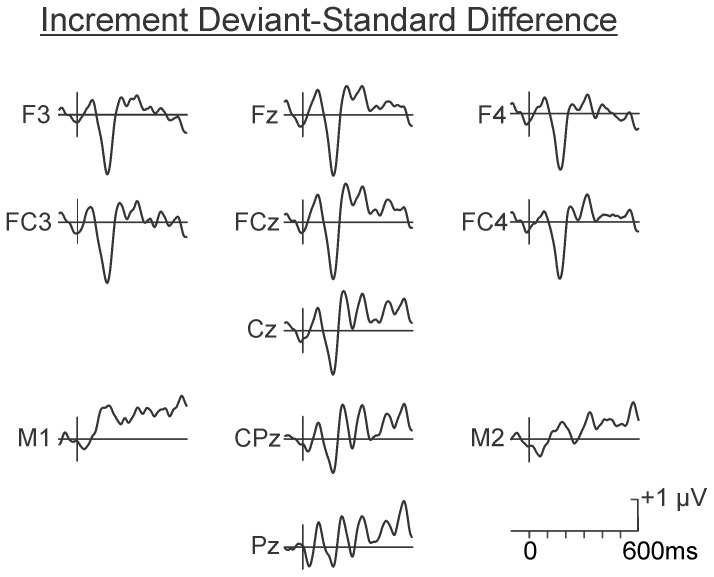
Grand average difference waves in the psychological increment condition. A large amplitude front-central maximum MMN occurring between 150 and 200 ms was evident following presentation of the psychological increment. A significant P3a-like centro-frontal maximum positivity peaking between 200 and 250 ms was also evident in the grand average waveform.

**Figure 6:**
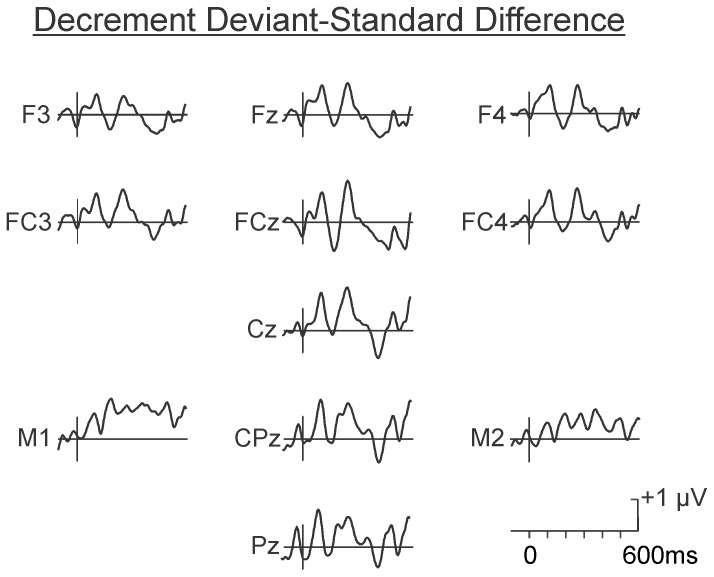
Grand average difference waves in the psychological decrement condition. The psychological decrement elicited a smaller non-significant negativity corresponding to the typical scalp distribution and latency of an MMN. No P3a-like positivity was apparent.

The difference waves in the psychological increment and decrement conditions are illustrated in Figures 5 and 6, respectively. As may be observed, the deviant in the increment condition (60-80-60-80-**80**-80) elicited a large MMN at Fz (M = −1.90 µV, SD = 1.92 µV), peaking at approximately 160 ms. Confidence interval testing indicated its amplitude differed significantly from the zero-voltage baseline, p<0.05. The MMN inverted in polarity at the mastoids. The deviant in the decrement condition (90-80-90-80-**80**-80) elicited a small MMN at Fz, peaking at approximately 220 ms. This small amplitude MMN was not significantly different from zero.

**Figure 7:**
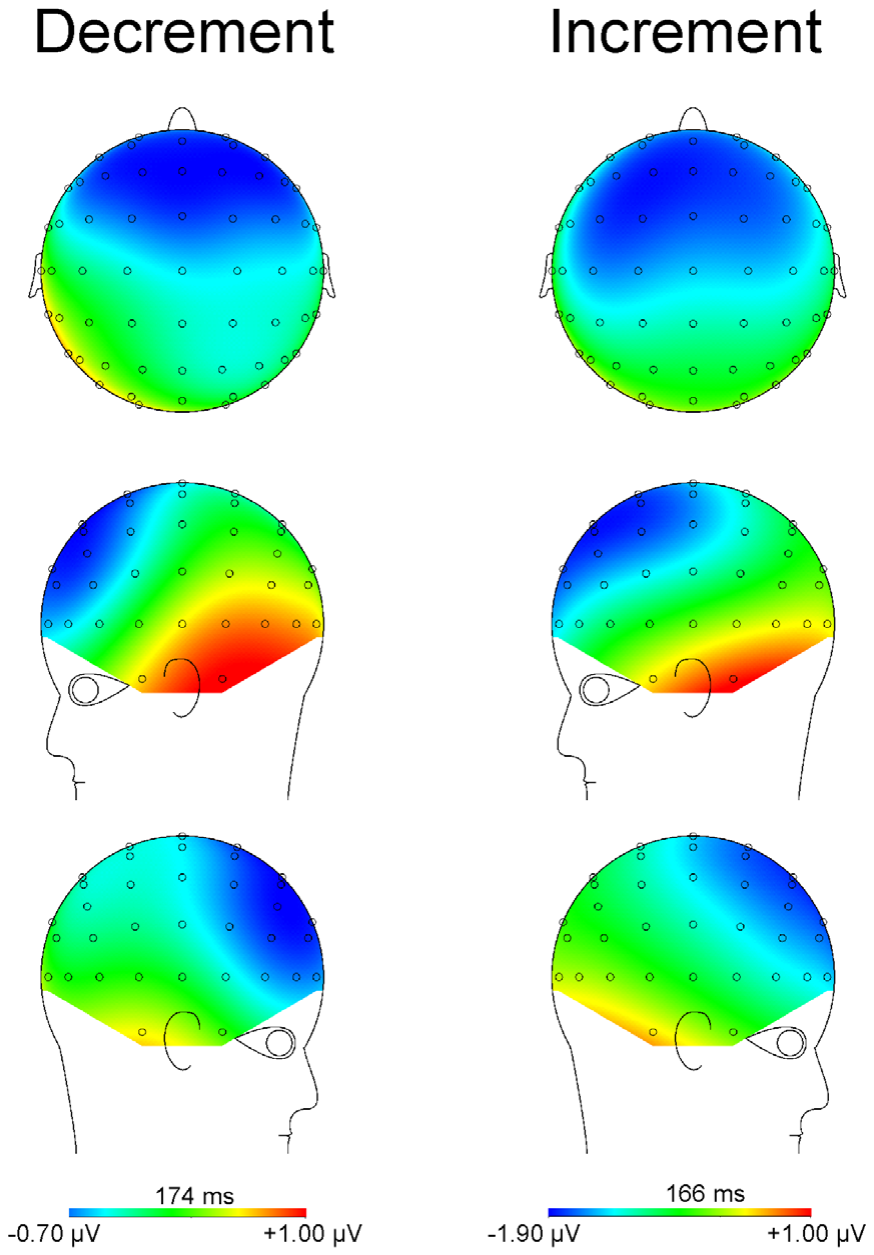
Spline scalp distribution maps of the MMN following presentation of the psychological decrements and increments. Although the MMN elicited by the psychological decrement did not attain significance, its scalp distribution was very similar to that elicited by the psychological increment. The MMNs were maximum over fronto-central areas and inverted in amplitude over inferior-lateral regions.

The scalp distribution of the small negativity in the decrement condition did however correspond to that of a true MMN, even though it did not differ significantly from the zero voltage pre-stimulus baseline level. The spline scalp distribution maps in the decrement and increment conditions are illustrated in Figure 7. As may be observed, the maps of the MMN elicited by the 80 dB SPL psychological decrement and the 80 dB SPL psychological increment are very similar.

Statistical analyses of the MMN were therefore carried out to compare its amplitude in the two conditions. A region of interest (ROI) analysis with the fronto-central electrode sites (Fz, F3, F4, FCz, FC3, FC4) was used. Amplitude data at these sites were subjected to an ANOVA with repeated measures on condition (increment, decrement), electrode region (anterior, posterior), and hemisphere (left, right). A significant main effect of condition was revealed for the amplitude of the MMN, F (1,8) = 5.08, p<.05. Increment deviants elicited a significantly larger amplitude MMN than decrement deviants. No main effects or interactions involving either anterior-posterior or inter-hemisphere electrode site were found, F<1 in all cases. A one-tailed t-test was used to compare the peak latency of the MMN response (at Fz) in the psychological increment and psychological decrement conditions. The latency of the MMN response was significantly earlier in response to the deviant in the psychological increment condition than the psychological decrement condition, t(8) = 1.93, p<.05.

The MMN was followed by a positivity, a P2/early P3a, at about 230 ms in both the psychological increment and decrement conditions. Its amplitude at Cz did not significantly differ between the two conditions, t<1. A second, centro-frontal maximum (late P3a) positivity peaking at approximately 320 ms was also apparent but only following presentation of the psychological increment. The amplitude of this positivity did not however significantly differ from the zero voltage pre-stimulus baseline level, p>.05.

### Discussion

The results of Experiment 2 largely replicate those of Experiment 1. Thus, the amplitude of the MMN was significantly larger and its latency significantly earlier to the psychological increment than to the psychological decrement deviant. Indeed, in contrast to the psychological increment, the negativity elicited by the psychological decrement failed to reach statistical significance from the zero voltage pre-stimulus baseline level when confidence interval testing was applied. The larger MMN in the increment condition could not be attributed to a smaller N1 to the standard in this condition. In fact, the standard N1 was larger in the psychological increment than in the psychological decrement condition, even though the intensity of the standards in both conditions were physically identical. The difference in N1 might be related to the relative perceptibility of the standards in the different conditions. The 80 dB SPL standard in the increment condition alternated with a 60 dB SPL tone and thus was more intense. The 80 dB SPL standard in the decrement condition alternated with the 90 dB SPL tone and was less intense.

The deviant in both increment and decrement conditions elicited an early positivity between 200 and 250 ms. This might correspond to a P3a. Its latency was, however, shorter than is typically associated with the P3a. Moreover, it is unlikely that a P3a would have been elicited in the psychological decrement condition when in the same condition, a significant MMN was not elicited. Some authors distinguish between an early and late P3a. The early positivity might be the first peak of a biphasic P3a similar to what has been observed in oddball paradigms [8]. It is however possible that this early positivity reflects P2 activity. Distinguishing between P2 and early P3a on either the basis of their scalp distribution or their functional significance has proven to be difficult [28]. Ceponiene et al. [29] have suggested that the early P3a might reflect the preparation for a possible attentional switch, as indexed by the late P3a. There was no evidence of the late P3a for the psychological decrement. As in Experiment 1, a late but non-significant P3a was apparent for the psychological increment.

## General Discussion

So critical is the detection of a change of an auditory stimulus that it occurs at various levels of the auditory system beginning as early as 10–40 ms after stimulus onset [30]. Detection of a change in the intensity of the stimulus is particularly relevant. Previous studies have indicated that in oddball paradigms, a deviant that represents a physical increment in intensity elicits a much larger DRN (probably a composite N1+ MMN) and P3a than a physical decrement. In the Näätänen model, this is probably because the increment results in greater activation of two different systems, the transient detector system (thus an increase in N1) and the change detection system (thus the MMN) than the standard, whereas the decrement results in only greater activation of the change detection system. The Macdonald and Campbell study [21] employed an alternating high and low intensity pattern, creating a deviant by the repetition of the standard. The psychological increment elicited a larger MMN and P3a than the psychological decrement and this was explained as a result of differential activation of only the change detection system. A problem with this interpretation was that the psychological increment and decrement deviants were also physically different, which may have confounded interpretation of the results. Experiments 1 and 2 of the present study employed physically identical standards and deviants. The psychological increment in Experiment 1 elicited a larger amplitude and shorter latency MMN than that elicited by the psychological decrement, mirroring the results of the Macdonald and Campbell [21] study. The time between the stimulus pairs in Experiment 1 was however short (100 ms) allowing for possible temporal integration of the intensity of the standard pairs. The intensity of these pairs varied between the increment and decrement conditions. Thus, it was possible that the larger MMN in the increment condition may still have represented a physical increase in intensity of the deviant pair (80–80 dB SPL) compared to the standard pair (80–60 dB SPL). In Experiment 2, the time between stimuli was much longer, 500 ms, removing the possibility of temporal integration. Again, the MMN to the psychological increment was significantly larger than that to the decrement. Similarly, the latency of the increment MMN was shorter, but the difference was not significant. Further, there was evidence of a P3a-like component in both experiments in response to the psychological increment, although these failed to reach statistical significance. The appearance of a P3a is thought to reflect a switching of attention from current cognitive activities to the potentially more relevant auditory channel (with more obtrusive stimuli eliciting larger P3as).

The present findings have been interpreted in the context of a violation of an alternating pattern rule (L follows H, H follows L), the violation of this rule leading to a psychological increment or decrement, respectively. As Horvath et al. [15] have pointed out, the alternating sequence might also establish two additional global-rule based streaming sequences, every other stimulus is low intensity (L-H-L) and every other stimulus is high intensity (H-L-H). The automatic perception of streaming does require a relatively rapid rate of stimulus presentation and a large difference among features [31], [32]. The difference in intensity of the two high and low intensity stimuli employed in this study was sufficiently large to permit easy detection of the pattern change and thus allow for perceptual streaming. The rate of stimulus presentation was however relatively slow in both experiments. The SOA between stimulus pairs was very long (1800 ms) in the first experiment and thus it was very unlikely that perceptual streaming could have occurred. If streaming had, in fact, occurred in either of the experiments, this should have resulted in a more perceptible H-H-H stream because of the higher intensity of these stimuli, causing this stream to “stand out” to the subject. Differences in MMN amplitude could thus be attributed to the differential perceptibility of the two streams. If this were the case, the MMN elicited in response to the deviant in the decrement condition (in which the deviant represents a disruption of the more readily perceived H-H-H stream) should have been larger than that elicited in the increment condition (in which the deviant represents a disruption of the less readily perceived L-L-L stream). This was, however, not the case. The deviant in the decrement condition resulted in a *smaller* amplitude MMN than that elicited in the increment condition.

The differential processing of the psychological increment and decrement deviants in Experiment 2 cannot be attributed to either differences in the physical parameters of deviant stimuli or the effects of temporal integration or perceptual streaming. The consistently larger MMN and subsequent P3a-like component to both physical and psychological increments thus present convincing evidence of the greater biological relevance of these auditory stimuli. The findings of the present experiments suggest this increased salience is not solely afforded to stimuli representing a *physical* increment relative to the immediate auditory past and thus activating both the change and transient detection systems. It is also afforded to any auditory deviant that violates an expectancy for a lower intensity stimulus, even if this deviant is physically identical to the immediate auditory past and thus results in increased activation of only the change detection system.
